# Contraindications to kidney transplantation: uneven grounds?

**DOI:** 10.1186/s13737-015-0024-x

**Published:** 2015-07-21

**Authors:** Bryce A. Kiberd, Meteb M AlBugami, Romuald Panek, Karthik Tennankore

**Affiliations:** Department of Medicine, Dalhousie University, Queen Elizabeth II Health Sciences-VG Site, Room 5082 Dickson Building, 5820 University Avenue, Halifax, B3H 1V8 NS Canada; Multiorgan Transplant Center, King Fahad Specialist Hospital, Dammam, Saudi Arabia

**Keywords:** Wait list, Eligibility, Guidelines, Kidney transplantation, Contraindications, Mortality, Dialysis

## Abstract

**Background:**

Determining eligibility for a kidney transplant is an important decision. Practice guidelines define contraindications to transplantation; however many are not evidence based. Canadian guidelines recommend that patients unlikely to survive the wait period not be evaluated. The purpose of this study was to evaluate what proportion of patients with a contraindication would survive the wait time.

**Methods:**

Consecutive incident dialysis patients (January 2006 to December 2012) with a contraindication, defined using Canadian guidelines, were studied. Mortality rates were determined for each individual contraindication. Theoretical survival to the median wait time to transplantation was calculated.

**Results:**

Of 746 incident patients, 435 (58 %) were deemed to have a contraindication at dialysis start. Nearly 80 % had a contraindication with a high mortality rate (dementia, multisystem disease, etc.). Patients with high mortality rates were less likely to survive the wait list than be transplanted. Patients with non-adherence, obesity, and potentially reversible disease had relatively low mortality rates, were more likely to survive, and possibly be transplanted at a time with the prospect of a better outcome.

**Conclusions:**

This study gives some credence that many patients with a contraindication are not likely to benefit. A better framework of defining contraindications is needed to allow better decision-making.

## Background

Eligibility for a kidney transplant is an important area of concern for patients with end-stage renal disease (ESRD). In a recent publication, Canadian Blood Services demanded that organ donation and transplantation services be fair, accountable, and transparent with regard to eligibility for the wait list and allocation of organs [1]. A recent review of worldwide practice showed considerable variability with regard to eligibility criteria and an overall concern for a lack of evidence-based decisions [2]. There is evidence that access to the list is associated with age, health literacy, race/ethnicity, socioeconomic status, co-morbidity, and region [3–10]. Many of the studies use large administrative datasets but these do not take into account the specific reasons for non-referral or ineligibility.

The Canadian Society of Transplantation set out eligibility criteria in 2005 [11]. These were mostly opinion based and have not been tested empirically. The rationale behind the definition of a contraindication to transplantation is variable. For example, immunosuppression in patients with active infection or cancer may cause greater harm. Some contraindications are potentially reversible (non-adherence, active kidney disease, etc.), and a delayed period of time might allow for a better relative outcome. Other conditions may not be true contraindications but rather may result in only marginal benefit for the patient (for example, transplanting patients with a projected short life expectancy).

A randomized control trial to test which contraindications result in more harm than benefit after transplantation would be required to provide high-quality evidence but is impractical if not impossible. It is likely that many not referred or even those with relative contraindications would benefit if there were a larger pool of available organs. It is also important to recognize that some patients are harmed with a transplant. Evidence shows that it may take a year or more before transplantation confers a survival advantage compared to waiting on the transplant list. The time to equal survival is dependent on the organ type, patient baseline mortality rate, and duration and magnitude of the increased risk early after the transplant procedure and start of immunosuppression [12].

One of the principles included in the Canadian eligibility document was that patients unlikely to survive the wait period not be evaluated [11]. Although low probability of surviving the wait period is not a contraindication to transplantation, it does address the issue of efficiency and whether resources should be used to evaluate patients who are not likely to benefit. The purpose of this study was to determine, based on the specific contraindication at the time of dialysis, what proportion would likely experience ‘no benefit’ from transplantation (as defined by death on the waitlist) or would potentially benefit (proportion surviving wait time and living beyond time of equal survival after transplantation) had they been listed and eligible for a kidney transplant despite their health status.

## Methods

Our center (Capital Health Authority) provides pre-dialysis and ESRD care including transplantation to an overall population of approximately 800,000 individuals from mainland Nova Scotia. The population sampled was a retrospectively analyzed cohort of all consecutive incident dialysis patients from January 2006 to December 2012 at this center. The sample contained 437 patients from a previous publication and an additional 309 patients (March 2011 to December 2012) [13]. Patients with a failed transplant were also considered in this analysis. The study was approved by our local research board (Capital Health Research Ethics Board).

As described in an earlier publication, eligibility for a kidney transplant was determined based on Canadian Society of Transplantation criteria as interpreted by the authors [13]. Categories of contraindication included dementia, multisystem disease (two or more contraindications), cancer, pulmonary disease (COPD), congestive heart failure (CHF), cardiovascular disease (CVD, including peripheral vascular disease and stroke), active kidney disease, active infection, active gastrointestinal (GI) issues (such as bleeding), obesity (body mass index (BMI) >40 kg/m^2^), non-adherence, and patient refusal. Patients were followed until June 2014. Electronic hospital records were used to identify contraindications and comorbidities (which were not necessarily contraindications), calculate the Charlson comorbidity index [13], and collect laboratory values at the start of dialysis [14]. This analysis was based on characterization at initiation of dialysis. However, if a contraindication was reversible and the patient was transplanted, the subject was still considered within the contraindication group but survival was censored at the time of transplantation.

To calculate the proportion that died on the wait list, we used median Canadian National time to transplantation data [15]. Many but not all centers require a patient to be on dialysis before listing. In this analysis, time zero was the start of dialysis rather than the time of pre-emptive wait listing. Since there is considerable variation between provinces, the shortest and longest median time were also analyzed. The Canadian National median time to transplantation is 1382 days (3.79 years) whereas the range among the provinces varied from shortest in Saskatchewan at 882 days (2.41 years) to the longest in British Columbia at 1954 days (5.34 years). Other provinces and their wait times were as follows: Alberta 1265 days, Ontario 1598 days, Quebec 1028 days, Manitoba 1861 days, and Atlantic Canada (including Nova Scotia) 981 days. The proportion that survive to transplantation was determined from the logarithmic survival function defined by proportion alive = exp^(-MR*t), where MR is mortality rate and *t* is time. In a sensitivity analysis, the proportion that survived was estimated by examining the Kaplan-Meier survival curves for the different groups of patients and visual comparison of the logarithmic survival function and Kaplan-Meier survival curves for differences in true and predicted cumulative survival. To estimate the proportion who survived to net benefit, the total time included median wait time and the time to equal survival. The time to equal survival after transplantation can vary from 120 days to over 750 days depending on the organ used (standard compared to expanded criteria) and patient mortality rate. As a conservative estimate, patients with a mortality rate of <20 deaths per 100 patient years were assumed to have a time of equal survival of 180 (0.5 years) days and those with a mortality >20 deaths per 100 patient years were assumed to have a time of equal survival of 365 (1 year) days [12]. Although it is likely that the time to equal survival might be considerably longer than 1 year for many of our patients with contraindications, there was limited published data to justify lengthening this time. If longer times are likely, then the proportion that would benefit from transplantation would be reduced.

Data are presented as means and standard deviation, median, and inter-quartile range or percentages where appropriate. Crude mortality rates with 95 % confidence intervals (CI) were determined by the number of deaths divided by years of exposure. Patient survival was examined by the Kaplan-Meier product limit method. A *p* value of <0.05 was the threshold for statistical significance. Statistical analysis was performed using Stata Version 12.0 (Texas, USA).

## Results

There were 746 adult patients that started dialysis (median follow-up of 2.13 (0.96, 3.6) years). Of these, 435 (58 %) were determined to have a contraindication at the start of ESRD treatment. Table 1 shows the characteristics and selected comorbidities of patients with a contraindication. There were 257 deaths (59 % of this cohort) with a crude mortality rate of 22.4 (95 % CI 19.7–25.1) deaths per 100 patient years. Reasons for contraindication, proportion of subjects within each contraindication category, mortality rate, probability of waitlist survival, and probability of survival to net benefit are shown in the Table 2. Nearly 80 % (78.4 %) of patients were in groups with <50 % probability of surviving the wait time and 36.6 % of the patients had a <25 % chance of benefiting from a transplant using the national median wait time average.

**Table 1:** Patient characteristics and selected co-morbidities

	Contraindication	Eligible and referred	Eligible but not referred	*p* value
	(*n* = 435)	(*n* = 215)	(*n* = 96)	
Age, years	65 ± 18	50 ± 15	74 ± 9	<0.001
Male, *n* (%)	269 (62)	134 (62)	55 (57)	0.63
Diabetes mellitus, *n* (%)	188 (43)	50 (23)	37 (39)	<0.001
Co-morbidities				
IHD, *n* (%)	181 (41)	29 (13)	25 (26)	<0.001
CHF, *n* (%)	137 (31)	20 (9)	20 (21)	<0.001
COPD, *n* (%)	91 (21)	17 (8)	23 (24)	<0.001
BMI, kg/m^2^	30 ± 9	29 ± 7	29 ± 6	0.038
Hemoglobin, g/L	96 ± 17	103 ± 19	101 ± 16	<0.001
eGFR (ml/min/1.73 m^2^)	8.5 ± 3.8	8.2 ± 3.3	10.5 ± 13.3	<0.001
Albumin, g/L	30 ± 6	35 ± 6	32 ± 5	<0.001
Charlson comorbidity index	5.4 ± 2.4	3.0 ± 1.8	4.5 ± 2.0	<0.001
Failed transplant, *n* (%)	32 (7)	14 (7)	2 (2)	0.163

^a^Continuous values are reported as mean ± standard deviation

**Table 2:** Probability of wait list survival and survival to benefit

Contraindication	Number	Mean age	Deaths	Deaths per 100 patient years	Probability of surviving	Probability of benefit, %
	(%)	(years)		(95 % CI)	Wait list, % (provincial range)	(provincial range)
Dementia	10 (2)	76	7	49.7 (23.7–104.2)	15 (7, 30)	9 (4, 18)
Multisystem	34 (7.8)	67	28	42.1 (29.1–61.1)	20 (11, 36)	13 (7, 24)
Cancer	74 (17)	68	56	35.9 (27.6–46.7)	26 (15, 42)	18 (10, 30)
CHF	42 (9.6)	69	26	29.7 (20.2–43.7)	32 (21, 49)	24 (15, 36)
Active kidney disease	32 (7.3)	64	18	27.6 (17.3–43.8)	35 (23, 51)	27 (18, 39)
CVD	140 (32)	68	89	22.7 (18.4–27.9)	42 (30, 58)	34 (24, 46)
COPD	10 (2.3)	73	7	20.6 (9.8–43.1)	46 (34, 61)	37 (27, 50)
Infection	11 (2.5)	61	6	16.5 (7.4–36.7)	54 (42, 67)	45 (35, 57)
GI	9 (2)	53	4	14.8 (5.6–39.4)	57 (46, 70)	54 (39, 65)
Refused	13 (2.9)	58	4	8.6 (3.2–23.0)	72 (63, 81)	70 (58, 78)
Obesity	19 (4.3)	57	4	5.4 (2.0–14.4)	82 (75, 88)	80 (71, 86)
Non-adherent	41 (9.4)	50	8	5.1 (2.5–10.2)	82 (76, 88)	80 (73, 86)

Some of the patients had a contraindication that was reversible (infection, gastrointestinal bleeding, etc.) or potentially reversible (obesity and non-adherence). Most of the patients with multisystem disease, cancer, and cardiovascular and chronic obstructive pulmonary disease had conditions that were irreversible. Table 3 shows the subset of patients that were felt to have irreversible disease with these conditions. Overall, this group (*n* = 275) suffered 206 deaths with a rate of 32 (95 % CI 27.8–36.6) deaths per 100 patient years. Of the 435 patients with a contraindication, 106 (24 %) were referred to the transplant team at some point over their care, 31 (7.1 %) were actually activated on the wait list after resolution of their contraindication, and 18 (4.1 %) were transplanted. Those listed included CVD-6, active kidney disease-5, non-adherence-8, multisystem-3, infection-2, cancer-3, GI-2, and refused-2. For example, patients with prior cancer that was in remission for the recommended wait time, corrected cardiovascular disease, improved adherence, resolution of infection, and correction of GI bleeding were activated.

**Table 3:** Mortality in patients with permanent contraindications

Contraindication	Number	Mean age (years)	Deaths	Deaths per 100 pt years	Probability of surviving wait list (%) (provincial range)	Probability of benefit (%) (provincial range)
Dementia	10	76	7	49.7 (23.7–104.2)	15 (7, 30)	9 (4, 18)
Multisystem	31	71	28	47.5 (32.8–68.7)	16 (8, 31)	10 (5, 20)
Cancer	67	67	54	38.8 (29.7–50.6)	23 (13, 40)	15 (9, 26)
CHF	36	68	25	34.8 (23.5–51.5)	32 (15, 43)	18 (11, 30)
CVD	121	71	85	26.0 (21.0–32.1)	43 (25, 53)	29 (20, 41)
COPD	10	73	7	20.6 (9.8–43.1)	46 (34, 61)	37 (27, 50)

**Fig. 1:**
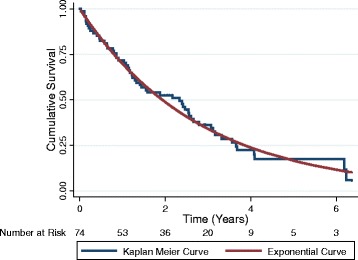
Cumulative survival for patients with a contraindication to transplantation due to cancer

**Fig. 2:**
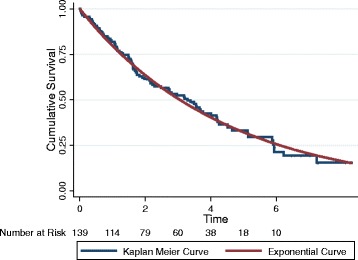
Cumulative survival for patients with a contraindication to transplantation due to coronary artery or vascular disease

**Fig. 3:**
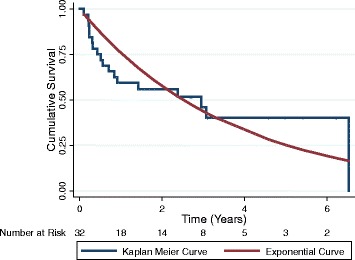
Cumulative survival for patients with a contraindication to transplantation due to acute kidney injury

Most survival rates approached a logarithmic decay (Fig. 1 for cancer and Fig. 2 cardiovascular disease). The differences between cumulative survival as evaluated by the Kaplan-Meier method and logarithmic function were minimal for most groups. The exception was the group with active kidney disease as shown in Fig. 3. This group had a very rapid death rate over the first year followed by a much lower mortality rate. The probability of waitlist survival and probability of survival to net benefit using life table analysis are shown in the supporting information file.

**Fig. 4:**
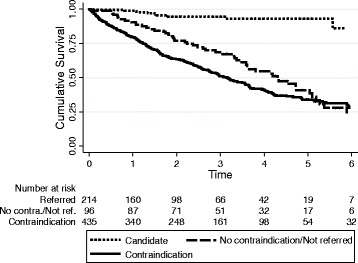
Cumulative survival in groups with a contraindication, candidates for transplantation and those with no contraindication but not referred

In the overall cohort of 746 patients, 215 (32 %) were referred for transplantation and had no contraindication at the start of dialysis (Table 1). These patients were younger with few co-morbidities (lower Charlson comorbidity index). The survival of this cohort was extremely high (only 10 deaths) with a crude mortality rate of 2.0 (95 % CI 1.0–3.7) deaths per 100 patient years (Fig. 4). Patients with this overall mortality rate were projected to achieve a survival benefit >90 % of the time. Table 1 and Figure 4 also show the remaining subset of 96 patients that were eligible but never referred. They were older with significant comorbidity and had a much higher overall mortality rate compared to eligible waitlisted patients (54 deaths, 17.2 deaths per 100 patient years, 95 % CI 13.2–22.5).

## Discussion

Much has recently been written on the principles of eligibility and organ allocation from a community, patient, and physician perspective [16–19]. Invariably, there are competing ethical principles that balance fairness, efficiency, and appropriate use of a scare resource. The aim of this study was to determine if patients with a contraindication were unlikely to survive the wait time to receive a deceased donor organ. The study shows that nearly 60 % of patients have a contraindication to transplantation at the start of dialysis. Most of these have a high mortality rate and would not survive long enough to benefit from a kidney transplant, especially those with an irreversible contraindication. There is considerable variation in mortality rates among the contraindication groupings.

The novel aspect of this study was that the reasons for the contraindications were determined by detailed patient chart review rather than using association data from administrative databases. The analysis did not use age or Charlson comorbidity burden as criteria for contraindication. As shown in an earlier study, older patients without contraindications that are not referred constitute a minority (<15 %) of incident population and tend to have relatively high mortality rates [14].

Although this study demonstrated that many contraindications were associated with a very low probability of waitlist survival or probability of surviving to a net benefit, it is important to note that kidney transplantation was not necessarily a futile option (<1 % benefiting) for many patients. At what point a condition should be deemed a contraindication based on likelihood of survival to benefit is unclear. Some would argue, including many patients, that any realistic possibility should be considered. This analysis simply presents an estimate of this proportion without necessarily making a final judgment. Some centers with long waiting times delay transplant eligibility evaluation until patients have sufficient wait time. For patients with high mortality contraindications, most will die prior to evaluation. In centers with relatively short transplant wait times, it is possible that more marginal candidates will undergo a transplant workup that may be prolonged and expensive.

Several groups with relatively low mortality rates deserve mention. Patients with obesity were a relatively small percentage of the cohort (4.3 %) and even smaller (2.6 %) when considering the entire population. Mortality rates were low in this group but numerically higher than waitlisted patients. Given the small numbers, a detailed analysis was not performed. Many registry studies treat obesity as a contraindication at a BMI >35 mg/kg^2^ [20]. Our local practice uses a higher cut-point. It is not clear whether obesity defined as a BMI >40 mg/kg^2^ is a legitimate contraindication [21].

A small percentage (<2 % of total cohort) refused to be transplanted and were included as a contraindication. Of the 13 patients, 8 had been referred but most did not complete their evaluation testing. The majority were male (69 %) with a mean age of 58. Patient refusals would not be captured in registry analyses. The causes of refusal are likely multifactorial and were not evaluated formally [22].

Non-adherence was also associated with a low mortality rate and comprised 5.4 % of the total incident population. Most of these patients were young. Of this group, 22 of the 41 were eventually referred to the wait list. Since graft loss and return to dialysis is associated with an increase in mortality, it could be argued that a trial of demonstrated adherence in subjects with a low mortality rate might be justified versus immediate transplantation [23]. Since our center dates wait time to dialysis start rather than to the date of listing, once on the wait list time has not been lost.

On the other hand, the group with active kidney disease had a very high mortality rate within the first year but then mortality rates were very low thereafter. Avoiding this high-risk period seems intuitively obvious for several reasons, including avoiding the risk of disease occurring in the transplanted kidney and optimizing a patient’s health status in those with active systemic disease. As with some of the stated contraindications, outcomes might be better if the contraindication was reversed before transplantation.

It should be pointed out that the analysis was likely to be optimistic in favor of transplantation. We did not specifically analyze the use of expanded criteria donors which would be associated with longer times to equal patient survival [12]. It is also likely that patients with higher mortality rates (>30 deaths per 100 patient years) would have even longer times to equal survival. If the time to equal survival was 2 years rather than 1 year, the probability of surviving to benefit would be reduced and could be estimated by multiplying the values in Table 2 by exp(-MR). For example, for CHF (MR = 0.297), the probability of survival to potential benefit would be reduced from 24 to 18 %. Prior studies calculating time to equal survival assume that patients on hold on the wait list have similar mortality rates to those who are active [24]. Eliminating hold patients would lengthen the time to equal survival and reduce the proportion likely to benefit [25]. Patients with contraindications included those that were reversible and those that were permanent. Permanent contraindication patients had much higher mortality. It was difficult to determine the exact timing for reversal of the contraindication, and analyzing this would introduce immortal time bias. Therefore, the analysis was based on the initial assessment of those likely to be permanent. Some but not all patients with reversible contraindications were referred. In some cases, a new contraindication developed in follow-up but this was not formally analyzed.

There are notable limitations to the study. This was a retrospective single center study, and there may well be regional variations in practice throughout Canada. There were relatively small numbers of Black or Native Canadian patients. All patients had medical coverage. Therefore, our population differs from the USA. However, the median time to transplantation in the US currently exceeds 1500 days for most adults [26]. Furthermore, current changes to the US allocation algorithm will reduce the number of older patients with co-morbidity from receiving a transplant which would increase wait times [27]. In this study, no patient was transplanted with an active contraindication; therefore, it is impossible to know the true risk or benefit. A better assessment of functional status including a quantitative scoring of frailty might also identify patients less likely to benefit. The study was not large enough to look at specific contraindications within groups (waiting 2 years for renal cell cancer after a cancer compared to 5 years for breast cancer). Given the length of follow-up and small numbers in some groups, survival was projected rather than using actual survival by life tables. The results of Table 2 using life table analysis are shown in the supporting information file. Some also would argue that quality of life was not factored in, but most of this time was spent on dialysis. Those who survived to transplantation would have experienced the pain of the operation and lived through an outpatient recovery time that may have been complicated and prolonged in this vulnerable population. These complications would also reduce the perceived benefits of transplantation.

Although formal statistics were not carried out prospectively, about 30 % of decisions required a third party review to determine whether a patient had a contraindication. One important area for further study would be a determination of inter-reviewer reproducibility for the determination of a contraindication as has been done in other studies [28]. There have been surveys examining attitudes of nephrologists towards transplant referral using case series but these have not applied a standard eligibility guideline [29, 30]. In addition, a several sentence description of a patient does not substitute for a full patient assessment of all the available data [28, 29]. A multicentre study evaluating the reproducibility of defining a contraindication with a follow-up educational tool that allows formal training in assessment would improve transparency and consistency for wait list eligibility determination on a national level.

Clearly, even patients with high baseline mortality are likely to be transplanted if they are listed prior to dialysis start, have a live donor, or are at centers with very short wait times. However, many Canadian centers do not list those not yet on dialysis. Patients with a live donor could potentially have much greater benefit, particularly if they are transplanted pre-dialysis before a contraindication develops. The larger issue is whether patients with increased baseline mortality are at a greater risk of early harm relative to uncertain later benefit with a transplant [25]. A significant risk of shortening a frail patient’s life associated with the transplant surgery and immunosuppression is a more compelling ethical rationale for not listing (do no harm) and certainly more defensible than either ‘not likely live long enough to be transplanted’ or ‘not likely to derive significant benefit’. Although there are no guaranteed outcomes, making common sense judgements with individual patients should prevail while encouraging further debate and study.

## Conclusions

For the most part, the current Canadian practice guideline defines contraindications to transplantation in patients with high mortality and low likelihood of surviving to transplantation or benefiting from this procedure. This provides some empirical evidence to a mostly opinion-based guideline. However, some contraindications are not associated with high mortality rates and are reversible. These patients are in the minority but are more likely to survive to have their contraindication reversed and subsequently receive a transplant at a time with the prospect of a better outcome. A better framework of defining contraindications and larger studies are needed to provide the granular detail not available in current registry analyses to allow better decision-making for clinicians.

## Abbreviations

ESRD: end-stage renal disease
BMI: body mass index
COPD: chronic obstructive pulmonary disease
CHF: congestive heart failure
CVD: cardiovascular disease
eGFR: estimated glomerular filtration rate
GI: gastrointestinal disease
